# African Swine Fever Vaccine Candidate ASFV-G-ΔI177L Produced in the Swine Macrophage-Derived Cell Line IPKM Remains Genetically Stable and Protective against Homologous Virulent Challenge

**DOI:** 10.3390/v15102064

**Published:** 2023-10-08

**Authors:** Manuel V. Borca, Ayushi Rai, Nallely Espinoza, Elizabeth Ramirez-Medina, Edward Spinard, Lauro Velazquez-Salinas, Alyssa Valladares, Ediane Silva, Leeanna Burton, Amanda Meyers, Cyril G. Gay, Douglas P. Gladue

**Affiliations:** 1 Plum Island Animal Disease Center, Agricultural Research Service, U.S. Department of Agriculture, Orient, NY 11957, USA; ayushi.rai@usda.gov (A.R.); nallely.espinoza@usda.gov (N.E.); elizabeth.ramirez@usda.gov (E.R.-M.); edward.spinard@usda.gov (E.S.); lauro.velazquez@usda.gov (L.V.-S.); alyssa.valladares@usda.gov (A.V.); amanda.meyers@usda.gov (A.M.); 2 National Bio and Agro-Defense Facility, Agricultural Research Service, U.S. Department of Agriculture, Manhattan, KS 66502, USA; ediane.silva@usda.gov (E.S.); leeanna.burton@usda.gov (L.B.); 3 Oak Ridge Institute for Science and Education (ORISE), Oak Ridge, TN 37830, USA; 4 Agricultural Research Service, U.S. Department of Agriculture, Beltsville, MD 20705, USA; cyril.gay@usda.gov

**Keywords:** ASFV, ASF, ASFV vaccine, ASFV-G-ΔI177L

## Abstract

ASFV vaccine candidate ASFV-G-ΔI177L has been shown to be highly efficacious in inducing protection against challenges with the parental virus, the Georgia 2010 isolate, as well as against field strains isolated from Vietnam. ASFV-G-ΔI177L has been shown to produce protection even when used at low doses (10^2^ HAD_50_) and shows no residual virulence even when administered at high doses (10^6^ HAD_50_) or evaluated for a relatively long period of time (6 months). ASFV-G-ΔI177L stocks can only be massively produced in primary cell macrophages. Alternatively, its modified version (ASFV-G-ΔI177L/ΔLVR) grows in a swine-derived cell line (PIPEC), acquiring significant genomic modifications. We present here the development of ASFV-G-ΔI177L stocks in a swine macrophage cell line, IPKM, and its protective efficacy when evaluated in domestic pigs. Successive passing of ASFV-G-ΔI177L in IPKM cells produces minimal genomic changes. Interestingly, a stock of ASFV-G-ΔI177L obtained after 10 passages (ASFV-G-ΔI177Lp10) in IPKM cells showed very small genomic changes when compared with the original virus stock. ASFV-G-ΔI177Lp10 conserves similar growth kinetics in primary swine macrophage cultures than the original parental virus ASFV-G-ΔI177L. Pigs infected with 10^3^ HAD_50_ of ASFV-G-ΔI177Lp10 developed a strong virus-specific antibody response and were completely protected against the challenge with the parental virulent field isolate Georgia 2010. Therefore, IPKM cells could be an effective alternative for the production of ASFV vaccine stocks for those vaccine candidates exclusively growing in swine macrophages.

## 1. Introduction

African swine fever (ASF) is a lethal disease that affects wild and domestic pigs and is widely geographically distributed in Europe, Africa, Asia, and the Caribbean area [1]. African swine fever virus (ASFV), the etiological agent of ASF, is a large, structurally complex virus with a large genome containing a double-stranded DNA of 180–190 kilobase pairs encoding more than 160 proteins [2,3].

Until recently, no commercial vaccines were available, and disease control was mainly performed by culling all the infected animals and severely restricting mobility of potentially susceptible animals.

In the last few years, several experimental live attenuated vaccine strains have been developed by deleting virus genes implicated in the process of ASFV virulence in pigs. Most of these attenuated strains were shown to be efficacious in inducing protection against the infection with the corresponding virulent parental field isolate [4,5,6,7,8,9,10,11,12,13].

One of these vaccine candidates is the ASFV-G-ΔI177L strain which has been shown to be highly efficacious in inducing protection against the challenge with either the parental Georgia 2010 isolate or the Vietnamese field isolate TKN/ASFV/DN/2019 [4,5]. ASFV-G-ΔI177L has been shown to efficiently induce protection without presenting residual virulence [14,15]. Production of ASFV-G-ΔI177L stocks requires the use of primary cell macrophage cultures. Adaptation to grow in swine cell line, although successful in terms of maintaining it safety and efficacy, produced undesirable significant genomic modifications. This type of genomic modification is a usual result in the process of the adaptation of an ASFV strain to grow in an established cell line [16]. We report here the production of ASFV-G-ΔI177L stocks using the swine macrophage cell line IPKM. The results demonstrate that ASFV-G-ΔI177L grows well in IPKM cells and that successive passages in those cells produce minimal changes in the genome of the obtained virus. Importantly, pigs infected with 10^3^ HAD_50_ of ASFV-G-ΔI177L passed 10 successive times in IPKM cells developed a strong virus-specific antibody response and conferred complete protection against the challenge with the parental virulent field isolate Georgia 2010. Thus, IPKM cells could be an effective alternative for the production of ASFV vaccine stocks for those vaccine candidates only growing in swine macrophages.

## 2. Materials and Methods

### 2.1. Viruses and Cells

ASFV-G-ΔI177L strain was previously developed in our laboratory [4]. Primary macrophage cell cultures were produced, as previously described [13], and they were seeded at a concentration of 5 × 10^6^ cells/mL. Virus titrations were performed using swine macrophage cultures, as previously described [13]. IPKM cells, immortalized porcine kidney macrophage-derived cell lines [17], were kindly provided by Dr Kokuho Takehiro from the National Institute of Animal Health of Japan. IPKM culture media was formulated exactly as previously described [17]. A series of 10 successive passages of ASFV-G-∆I177L in IPKM cell cultures were performed at an MOI of 1. Each infection step was allowed to proceed until reaching approximately 80% of the cytopathic effect, at which point cultures were frozen. Intermediate stocks were then prepared by thawing the cell culture suspension, clarification by centrifugation, and titration of virus suspensions on primary swine macrophage cultures, as described below. Comparative growth kinetics between the original ASFV-G-∆I177L stock (ASFV-G-∆I177Lp0) and the stock produced after 10 successive passages in IPKM cells (ASFV-G-∆I177Lp10) were set using an MOI of 0.01 HAD_50_, as previously described [13] with sample points obtained at 2, 24, 48, 72, and 96 h post-infection. Samples were titrated using swine macrophage cell cultures in 96-well plates. Virus-infected cells were detected by hemadsorption (HA), as described in chapter 3.9.1. of the *OIE Manual of Diagnostic Tests and Vaccines for Terrestrial Animals* [18]. Briefly, 20 μL of a fresh preparation of 1% pig erythrocytes in buffered saline was added to each well of the 96-well plate. The presence of rosettes was recorded for 7 days, and virus titers were calculated by the Reed and Muench method [19].

### 2.2. Sequencing and Analysis of the ASFV-G-∆I177L Genome

ASFV-G-∆I177Lp0 and ASFV-G-∆I177Lp10 DNA were extracted from the infected IPKM cells using a DNA extraction kit (Qiagen DNeasy Blood and Tissue Kit). DNA concentration was determined using the Qubit dsDNA high-sensitivity (HS) assay kit (Life Technologies; Carlsbad, CA, USA) and read on a Qubit 2 fluorometer (Life Technologies). The DNA library was then used for NGS sequencing using a Nextera XT kit in the NextSeq sequencer (Illumina, San Diego, CA, USA), strictly following the manufacturer’s protocol. Sequence analysis was performed using CLC Genomics Workbench software (CLCBio, Waltham, MA, USA).

Analysis of the virus genome was performed using CLC Genomics Workbench v23 (QIAGEN, Aarhus, Denmark). Illumina reads were trimmed for quality (limit = 0.05), ambiguous base pairs (max = 2), adapters, and minimum size (min = 50) and from the 5’ (20 nucleotides) and 3’ terminal end (5 nucleotides) and were mapped against ASFV Georgia 2007/1 (Genbank accession: FR682468.2) using the “Map Reads to Contigs” tool with the following parameters: contig Masking = off; update contigs = off; match score = 1; mismatch score = 2; gap cost = linear; insertion and deletion cost = 3; length fraction = 0.7; similarity fraction = 0.8; global alignment = off; auto-detect paired distances = on; and non-specific match handling = random. Basic Variant detection was then performed on the read mappings using the following parameters: ploidy = 2; ignore positions with coverage over 2,000,000; ignore broke pairs = off; ignore non-specific matches = off; minimum coverage = 1; minimum count = 1; minimum frequency = 50%; and filters for quality, direction/position, and technology specifics = off. Single nucleotide polymorphisms (SNPs) that appeared in over 70% of reads were considered to be of high confidence and were compared to the original batch of ASFV-G-∆I177L and reported.

### 2.3. Differential Detection of ASFV-G Genome in Challenge Animals

Real-time PCR (qPCR) was used to differentiate between the presence of the parental virus ASFV-G and ASFV-G- I177Lp10, as previously described [20]. As an overall marker for the presence of ASFV, the presence of p72 gene was used with primers and probes as in the standard diagnostic test for ASFV: forward 5’-CTTCGGCGAGCGCTTTATCAC-3′, reverse 5′-GGAAATTCATTCACCAAATCCTT-3‘, and probe 5′-6FAM-CGATGCAAGCT TTAT-MGB-NFQ-3’. For the specific detection of the portion of the I177L gene deleted from the ASFV-G-ΔI177L genome, the following primers were used: forward 5′-GAACTGGAAAAAACTTTAACGGC-3′; reverse 5′-CCATTACCGGCAAGCTAGG-3′; and probe 5′-6FAM-ACGGATCCCCCTTCGCATTTGA-MGB-NFQ-3’. These oligonucleotides target the I177L gene that is absent from the ASFV-G-ΔI177L genome.

### 2.4. Detection of ASFV Specific Antibodies

ASFV antibody detection was performed using an in-house ELISA, as previously described [21]. Briefly, ELISA antigen was prepared from ASFV-infected Vero cells. Maxisorb ELISA plates (Nunc, St Louis, MO, USA) were coated with 1 µg per well of infected or uninfected cell extract. The plates were blocked with phosphate-buffered saline containing 10% skim milk (Merck, Kenilworth, NJ, USA) and 5% normal goat serum (Sigma, Saint Louis, MO, USA). Each swine serum was tested at multiple dilutions against both infected and uninfected cell antigens. ASFV-specific antibodies in the swine sera were detected using an anti-swine IgG-horseradish peroxidase conjugate (KPL, Gaithersburg, MD, USA) and SureBlue Reserve peroxidase substrate (KPL, Milford, MA, USA). Plates were read at OD630 nm in an ELx808 plate reader (BioTek, Shoreline, WA, USA). Sera titers were expressed as the log10 of the highest dilution, where the OD630 reading of the tested sera at least duplicated the reading of the mock-infected sera.

### 2.5. Evaluation of ASFV-G-ΔI177Lp10 Efficacy in Domestic Pigs

The efficacy of ASFV-G-∆I177Lp10 in inducing protection against challenges with the parental virus African swine fever Georgia2010 isolate (ASFV-G) was assessed in experimentally infected 35–40 kg commercial breed pigs. Groups of pigs (*n* = 5) were intramuscularly (IM) inoculated with 10^3^ HAD_50_ of either ASFV-G-∆I177Lp10 or mock-inoculated. The appearance of clinical signs (such as depression, anorexia, staggering gait, purple skin discoloration, diarrhea, and cough), as well as changes in body temperature, were recorded daily for 28 days. Blood samples were scheduled to be obtained at days 0, 4, 7, 11, 14, 21, and 28 post-inoculation (pi). At day 28 pi, both groups of animals were IM challenged with 10^2^ HAD_50_ of ASFV-G. Animals were monitored and sampled as described above until day 21 post-challenge. All animal experiments were performed under biosafety level 3 conditions in the animal facilities at Plum Island Animal Disease Center, strictly following a protocol approved by the Institutional Animal Care and Use Committee (225.06-19-R_090716, approved on 6 September 2019).

## 3. Results and Discussion

### 3.1. Successive Passages of ASFV-G-ΔI177L in IPKM Cells

**Figure 1:**
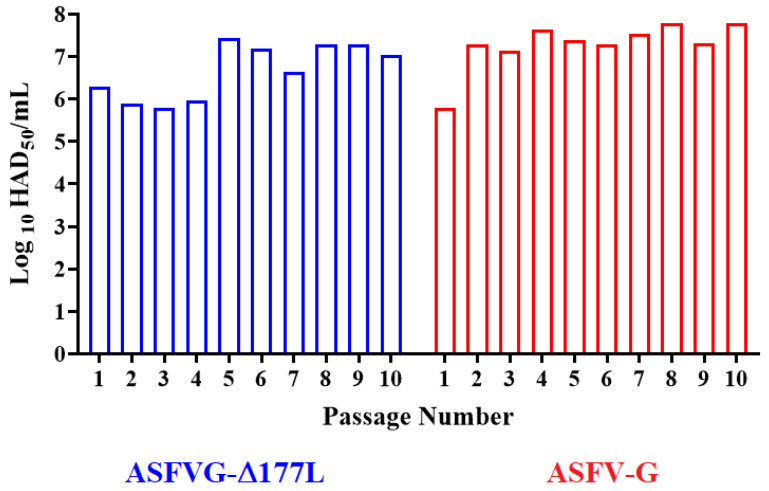
Replication of ASFV-G-ΔI177L in successive passages in IPKM cells. ASFV-G-ΔI177L and ASFV-G were passed 10 consecutive times (MOI = 1) in IPKM cell cultures. Viral yield in each passage was quantified in primary cultures of swine macrophages and titers expressed as HAD_50_/_mL_. Titrations were conducted by duplicated (presented data represent one set of them).

To assess the ability of IPMK to support virus growth without the need for an initial period of adaptation, the ASFV-G-∆I177L virus was successively passed 10 times in IPMK cells. All passage steps were conducted with an MOI of 1, based on stock titers calculated by titrations performed in primary macrophage cell cultures. Each infection step was allowed to proceed until reaching approximately 80% of the cytopathic effect, at which point cultures were frozen, intermediate stocks were prepared as described in Material and Methods, titrated, and prepared for the next passage. A parallel set of passages was also performed with a viral stock of the parental virus ASFV Georgia 2010 isolate (ASFV-G). The results demonstrated that replication of both ASFV-G-∆I177L and the parental ASFV-G remained relatively stable along the 10 passages (Figure 1). Variation in final titers between passages remained in a titer range of 10^5.8−7.3^ HAD_50_/_mL_ for ASFV-G-∆I177L and 10^5.8−7.8^ HAD_50_/_mL_ for ASFV-G. Therefore, it appears that both viruses efficiently replicate in IPKM cells without an obvious process of initial adaptation. These results corroborate previously published ones demonstrating that several ASFV strains can readily replicate in IPKM cells [22], where ASFV strains Armenia07, Kenya05/Tk-1, Espana75, and Lisbon60 were shown to replicate in IPKM as efficiently as they do in primary cultures of pulmonary-derived swine macrophages.

### 3.2. Genomic Modifications of ASFV-G-ΔI177L during Passages in IPKM Cells

After passage 4 and 10 in IPKM cells (ASFV-G-∆I177Lp4 and ASFV-G-∆I177Lp10, respectively), ASFV-G-∆I177Lp4 and ASFV-G-∆I177Lp10 were sequenced and compared to the original stock of the virus (ASFV-G-∆I177Lp0). A total of three mutations of high confidence (over 70% of the reads at that position contained the SNP) were observed. In ASFV-G-∆I177Lp4, two mutations were observed. The first, at position 115,987, within the DNA polymerase (gene G1211R) coding region, an A to C mutation did not result in a change to the amino acid sequence. The second mutation was within the coding region of the E199L inner virion membrane protein, where there was an A to T mutation that resulted in a serine-to-threonine mutation at position 133. While the mutation within E199L was maintained for all 10 passes of the virus in IPKM cells, the silent mutation within G1211R was no longer present after 10 passes. In addition, ASFV-G-∆I177Lp10 presented an insertion of a single A in a non-coding region at position 1362. Therefore, after 10 passages in IPKM cells, ASFV-G-∆I177Lp10 acquired only one amino acid mutation in the E199L gene. This gene encodes for a structural protein described as being involved in the processes of cell autophagia [23] and virus entry to the target cell [24]. It is not clear if the serine to threonine amino acid mutation found in the ASFV-G-∆I177Lp10 may affect the function of E199L protein in any of the above-described functions, particularly considering the similar characteristics of both amino acid residues. Similarly to these results, the stability of the genome sequence of the Armenia 2007 isolate after consecutive 15 passages in IPKM was also reported [22].

### 3.3. Assessment of Kinetic Replication of ASFV-G-ΔI177L in IPKM Cells

To evaluate the growth kinetics ability of ASFV-G-∆I177L on IPKM cells, a study was conducted where replication of the original ASFV-G-∆I177L (ASFV-G-∆I177Lp0) was compared to that of the virus after 10 passages in IPKM (ASFV-G-∆I177Lp10). ASFV-Gp0 and ASFV-Gp10 were added as control. A multistep growth curve was performed in primary swine macrophage cultures infected at a low MOI (0.01) with either ASFV-G-∆I177Lp0 and ASFV-Gp0 or their respective product obtained after 10 passages in IPKM cells (ASFV-G-∆I177Lp10 and ASFV-Gp10). Virus yields were evaluated at 2, 24, 48, 72, and 96 h post-infection by titration in primary swine macrophages.

**Figure 2:**
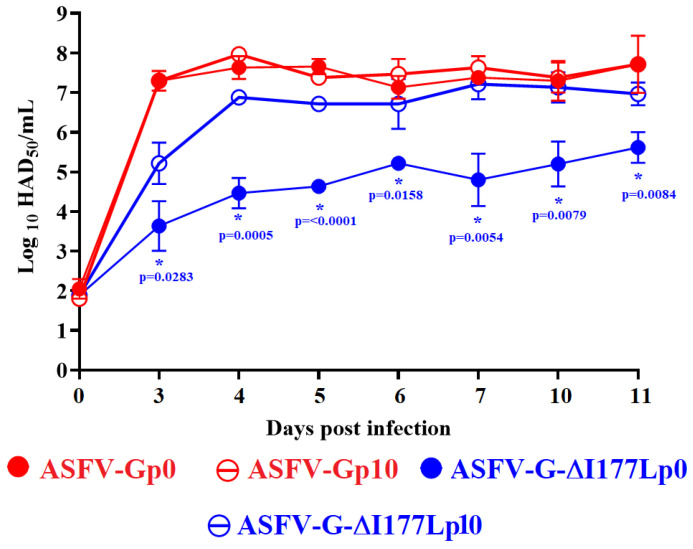
In vitro growth kinetics of ASFV-G-ΔI177Lp10 and parental ASFV-G-ΔI177Lp0 in primary swine macrophage cell cultures (MOI = 0.01). As control, ASFV-Gp0 and ASFV-Gp10 were tested under similar conditions. Samples were taken from two independent experiments at the indicated time points and titrated in swine macrophages. Data represent means and standard deviations. Sensitivity using this methodology for detecting virus is ≥log10 1.8 HAD_50_/mL. (*) Indicates significant differences. Statistically significant differences at specific time points between groups were evaluated by ANOVA analysis and confirmed by Tukey’s honest significance test (≤0.05). Analyses were conducted using the software JMP Pro version 16.0.0.

The results demonstrated that ASFV-Gp10 showed an almost indistinguishable kinetics of replication when compared to the parental ASFV-Gp0. No statistical differences were found in any of the time points tested (Figure 2). Conversely, ASFV-G-∆I177Lp0 exhibited a decreased replicative ability when compared with ASFV-G-∆I177Lp10, which presented a replicative kinetics comparable to that of the ASFV-Gp0 and ASFV-Gp10 viruses. Differences between ASFV-G-∆I177Lp0 and ASFV-G-∆I177Lp10 varied between 10^1.5^ to 10^2.5^ HAD_50_/_mL_, depending on the time point considered post-infection. Therefore, it appears that along the passages, ASFV-G-∆I177Lp10 acquired small mutations that may be responsible for increasing its ability to replicate in IPKM cells.

### 3.4. Assessment of ASFV-G-ΔI177Lp10 Replication in Domestic Pigs

**Figure 3:**
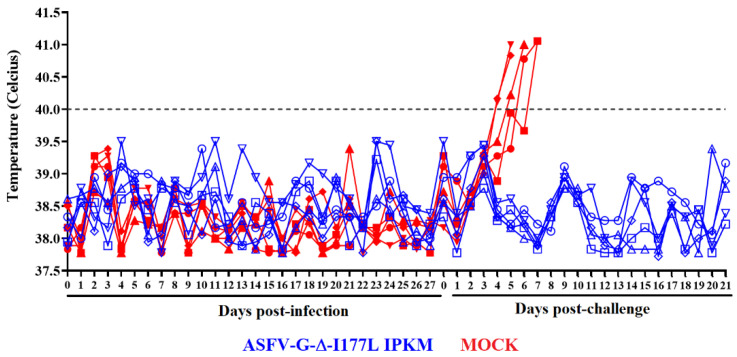
Evolution of body temperature in animals (5 animals/group) IM inoculated with 10^3^ HAD_50_ of ASFV-G-ΔI177Lp10 or mock-inoculated and challenged 28 days later with 10^2^ HAD_50_ of parental virulent ASFV-G. Data represent individual animals.

To evaluate the efficacy of ASFV-G-∆I177Lp10 in inducing protection in domestic pigs against infection with the parental virulent ASFV-G, a group (*n* = 5) of 35–40 kg pigs was inoculated IM at a dose of 10^3^ HAD_50_. This vaccine dose was selected because it resembles the dose of the virus recommended by the manufacturer of the commercial version of the ASFV-G-∆I177L produced in primary cultures of swine macrophages [5]. A control group of animals with similar characteristics was mock-inoculated. The appearance of clinical signs potentially associated with ASF was monitored daily for 28 days after inoculation. All animals in both groups remained clinically normal. The record of the rectal temperature values in animals of both groups remained within a normal range (below 40 °C) (Figure 3). Therefore, ASFV-G-∆I177Lp10 remains completely attenuated to domestic pigs when inoculated under the condition described here. 

**Figure 4:**
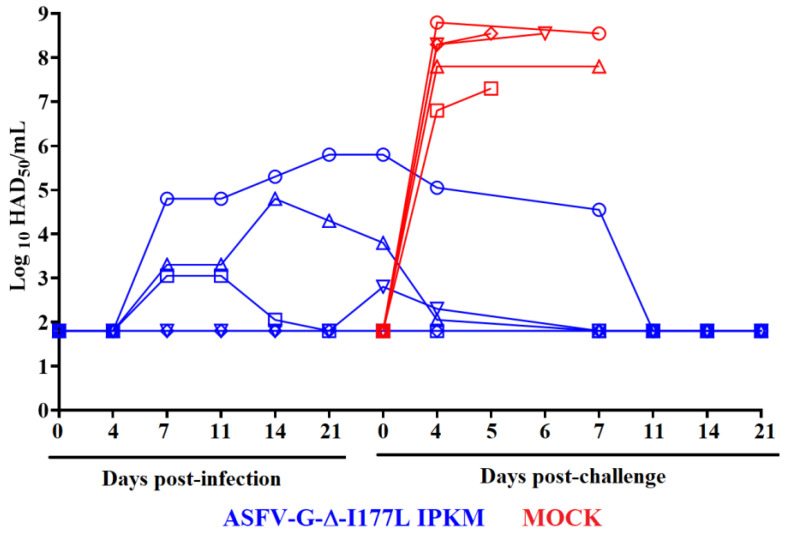
Viremia titers detected in animals (5 animals/group) IM inoculated with 10^3^ HAD_50_ of ASFV-G-ΔI177Lp10 or mock-inoculated and challenged 28 days later with 10^2^ HAD_50_ of ASFV-G. Data represent individual animals. Sensitivity of virus detection: ≥10^1.8^ TCID_50_/_mL_.

The replication of recombinant ASFV-G-∆I177Lp10 in the inoculated animals was analyzed by quantifying viremia titers at different times post-inoculation (pi). Viremia kinetics in these animals exhibited a heterogeneous pattern (Figure 4). One of the animals presented a detectable viremia at day 7 pi with a relatively high titer (approximately 10^5^ HAD_50_/_mL_) and showed similar viremia values (ranging from 10^4.8^–10^6^ HAD_50_/_mL_) until day 28 pi. Another two animals presented low viremia titers by day 7 pi (approximately 10^3^ HAD_50_/_mL_), and while one of them reached titer values ranging from 10^3.2^–10^5^ HAD_50_/_mL_ until day 28 pi, the viremia titers in the other animal fluctuated between being undetectable (≤10^1.8^ HAD_50_/_mL_) to 10^3.2^ HAD_50_/_mL_. Viremias in the remaining two animals were at undetectable levels (≤10^1.8^ HAD_50_/_mL_) in all tested time points.

The kinetics of replication exhibit lower viremia titers than those observed in pigs with similar characteristics inoculated IM with 10^2^ HAD_50_/_mL_ of the original stock ASFV-G-∆I177L (ASFV-G-∆I177Lp0) [4].

### 3.5. Assessment of the Efficacy of ASFV-G-ΔI177Lp10 to Protect Domestic Pigs against the Challenge with the Virulent Parental ASFV-G

Although the host immune mechanisms producing protection against infection with virulent strains of ASFV are still not well-defined [25,26], our previous experience indicated that the only parameter consistently associated with protection against challenge is the level of circulating antibodies [21]. Assessment of the presence of virus-specific antibodies in the ASFV-G-ΔI177Lp10-inoculated animals was detected in the sera of these animals using in-house-developed ELISAs [21]. 

**Figure 5:**
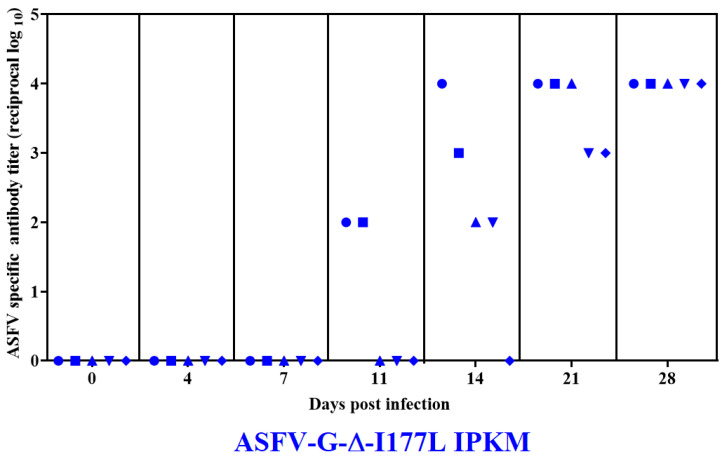
Anti-ASFV antibody titers detected by ELISA in pigs IM inoculated with 10^3^ HAD_50_ of ASFV-G-ΔI177Lp10. Each point represents values from individual animals. Titers are expressed as the log_10_ of inverse of the highest serum dilution that still duplicates OD of the pre-inoculation serum.

All animals infected with ASFV-G-ΔI177Lp10 developed a circulating antibody response, which was presented in the first two of the animals by day 11 pi, in all of them by day 21 pi, and reached the highest titers y day 28 pi (Figure 5). Therefore, all animals developed a strong antibody response comparable to that described in animals inoculated with IM containing 10^2^ HAD_50_/_mL_ of the original stock ASFV-G-∆I177L (ASFV-G-∆I177Lp0) [4]. No antibody titers were detected in the mock-vaccinated group at any time point tested.

To further assess the ability of ASFV-G-ΔI177Lp10 to induce protection against the challenge of highly virulent parental virus ASFV-G, the animals previously inoculated with 10^3^ HAD_50_/_mL_ of ASFV-G-ΔI177Lp10 were IM-infected at 28 days later with 10^2^ HAD_50_ of ASFV-G. The mock vaccinated group (*n* = 5) of naïve animals was included as the control group and was inoculated under the same conditions.

Mock animals started showing clinical signs of the disease by days 4–5 post-challenge (dpc), worsening in their clinical presentation quickly with animals being euthanized due to the severity of the disease: two of them by day 5; one on day 6; and the remaining two by day 7 pc (Figure 3). Conversely, animals in the group previously inoculated with ASFV-G-ΔI177Lp10 remained clinically normal during the 21-day observation period. Therefore, immunization with ASFV-G-ΔI177Lp10 induced solid protection against clinical disease after animals were challenged with the highly virulent parental virus.

Viremia titer values in the control animals after the challenge with ASFV-G peaked by the 4^th^ dpc (ranging between 10^6.8^ and 10^8.8^ HAD_50_/_mL_) and stayed at that level until all animals needed to be euthanized due to the severity of the clinical signs. Conversely, after the challenge, none of the animals previously inoculated with ASFV-G-ΔI177Lp10 showed viremia titers higher than those present at the time of the challenge. Actually, viremia titers in these animals gradually declined, with the viremia levels in all these animals being undetectable by the 11th dpc. (Figure 4).

To evaluate the presence of replication of the ASFV-G after the challenge in the protected animals, blood samples showing the highest viremia titers in each animal were then tested using specific real-time PCRs for the detection of p72-, I177L- and mCherry-genes following procedure previously described [20]. All positive samples detected the presence of p72 and mCherry genes but were negative for I177L, indicating the absence of challenge virus. These results indicate that replication of the challenge virus did not occur in all ASFV-G-ΔI177Lp10-infected animals. This result is similar to that obtained in animals immunized with ASFV-G-ΔI177L (ASFV-G-ΔI177Lp0) [4].

In summary, we showed here that the ASF live attenuated vaccine strain ASFV-G-ΔI177L could be grown for at least 10 successive passages in the swine macrophage-derived cell line IPKM acquiring minimal genetic modifications and be as safe and efficacious to protect animals against the infection with the virulent ASFV-G strain as the parental ASFV-G-ΔI177L is. 

These results open the possibility of using IPKM cells as subtract to grow ASFV vaccine strains originally produced and grown in primary swine macrophage cell cultures, which represents a limitation in the massive production of vaccine viruses for commercial purposes. The use of a cell line would also provide a contamination-free cell substrate and would avoid the systematic use of animals as cell providers. 

## Data Availability

All data are included in the manuscript.
